# The association between dietary protein intake and colorectal cancer risk: a meta-analysis

**DOI:** 10.1186/s12957-017-1241-1

**Published:** 2017-09-08

**Authors:** Renxu Lai, Zhuang Bian, Hong Lin, Jiangnan Ren, Huaili Zhou, Huixue Guo

**Affiliations:** Department of Gastroenterology, The Fifth Affiliated Hospital of Sun Yat-sen University, No.52, Meihua East Road, Zhuhai, Guangdong 519000 China

**Keywords:** Protein intake, Colorectal cancer, Meta-analysis, Relative risk

## Abstract

**Background:**

Association between dietary protein intake and colorectal cancer risk has not been fully quantified, while the results were controversial. This study aimed to evaluate the role of protein intake in the development of colorectal cancer.

**Methods:**

PUBMED and EMBASE were searched up to December 2016. Two independent reviewers independently extracted data from eligible studies. Relative risk (RR) with 95% confidence intervals (CI) was pooled using random-effects model to estimate the result. Besides, publication bias and sensitivity analysis were conducted.

**Results:**

Thirteen articles involving 21 studies comprising 8187 cases were included in this report. The pooled RR of colorectal cancer was 1.006 (95% CI = 0.857–1.179) indicating that there is no significant association between dietary protein intake and colorectal cancer risk. Furthermore, the pooled RRs for colon cancer and rectum cancer were 1.135(95% CI = 0.871–1.480) and 0.773(95% CI = 0.538–1.111), respectively, with the highest category of dietary protein intake. The association was not significant either in subgroup analysis of study design, protein type (animal protein or vegetable protein), sex, and or geographic locations.

**Conclusions:**

The present study indicated that the highest category compared to the lowest category of protein intake had no significant association on colorectal cancer risk. Dose-response analysis was not conducted due to limited information provided. Therefore, more studies with large cases and participants as well as detailed amounts of dietary protein intake are wanted to confirm this result.

**Electronic supplementary material:**

The online version of this article (10.1186/s12957-017-1241-1) contains supplementary material, which is available to authorized users.

## Background

Colorectal cancer is one of the high incidence of malignant tumors worldwide, with the morbidity and mortality ranking third among malignant tumors in the Western world [1]. Owing to economic development, air and water pollution, and changing lifestyle to high-protein, high-carbohydrate, and high-fat diet, the incidence and mortality of colorectal cancer has been on a rising trend in China [2]. In general, physical activity, low total energy intake such as dietary protein, low red and processed meat consumption, and limited alcohol drinking were known to give beneficial effect for cancer prevention [3]. Diet has long been regarded as the most important lifestyle risk factor for colorectal cancer. However, role of many dietary factors in colon carcinogenesis remains unresolved.

In animal and in vitro studies which investigated the effect of protein intake on colorectal cancer risk, high-protein diet could lead to DNA damage of colonocytes, decrease colonic mucosal thickness, and reduce the height of the colonocyte brushborder membrane [4–6]. Several epidemiologic studies have explored the relationship between dietary protein intake and the risk of colorectal cancer, but the results are inconsistent. A recent study by Tayyem RF et al. reported that the highest intake of protein could increase colorectal cancer risk [7]. However, Sun Z et al. found that colorectal cancer risk could be reduced with the highest category intake of protein [8]. Furthermore, Yang SY et al. failed to find any significant association between them [9]. So far, there is no meta-analysis conclusively demonstrating the relationship between them. In this study, a comprehensive meta-analysis of observational studies was performed to assess the colorectal cancer risk associated with dietary protein intake. The aim of this report was also to assess the heterogeneity and publication bias.

## Methods

### Search strategy

This meta-analysis followed the “Preferred Reporting Items for Systematic Reviews and Meta-Analyses” (PRISMA) guidelines [10]. We systematically searched PubMed and Embase (from their commencements to December 2016) for studies with the following format: (protein OR nutrition) AND (colorectal cancer OR colon cancer OR rectum cancer). In addition, the reference lists of relevant articles were also reviewed to find other suitable articles.

### Study selection

Studies were included if they met all of the following criteria: (1) the study subjects were adults (≥ 18 years old) without specific diseases at study baseline; (2) the study was conducted with observational studies; (3) the exposure of interest was dietary intake of protein with two or more quantitative categories; (4) the outcome of interest was colorectal cancer, colon cancer, or rectum cancer; and (5) relative risk (RR) and 95% confidence intervals (CI) for dietary protein intake (or there are sufficient data to compute them was also acceptable).

### Data exaction and quality assessment

The following data from each study were extracted independently by two authors: the first author’s name, publication year, study location, age and sex of the study population, outcomes of disease type, type of protein intake, number of cases and controls, variables adjusted in each original study, and RR with their 95% CI. If the paper reported total protein, animal protein, and vegetable protein at the same time, we used the data of the total protein for the whole analysis and animal protein or vegetable protein for the subgroup analysis if possible. Otherwise, animal protein and vegetable protein was as an independent study for the whole analysis. Furthermore, sex (male or female) and disease type (colorectal cancer, colon cancer, or rectum cancer) was analyzed as the same as protein type. Newcastle-Ottawa Scale (NOS) was used to assess the quality of included studies [11], which can either be used as a checklist or scale. The full score was nine stars, and a study with ≥ 6 stars was defined with high-quality.

### Statistical analysis

The pooled RR with 95% CI was calculated using random-effects models with the method of DerSimonian and Laird, which considers both within-study and between-study variation [12]. The between-study heterogeneity in the association between dietary protein intake and colorectal cancer was assessed using chi-square test and *I* ^*2*^ test, and < 25%, 25–50%, and > 75% represents low, moderate, and severe heterogeneity, respectively [13, 14]. Subgroup analyses were performed by disease type, study design, sex, protein type, and geographic locations. Sensitivity analysis was performed to assess whether the results could be affected while removing a single study [15]. The individual study is thought to produce excessive influence, if the point estimate lies outside the 95% CI of the combined analyses. Small study effect was assessed with visual inspection of the Egger’s test and funnel plot [16]. We used STATA version 12.0 (Stata Corporation, College Station, TX, USA) for the meta-analysis. Two-tailed *P* ≤ 0.05 was accepted as statistically significant for computed effects.

## Results

### Characteristics of studies and data quality

**Fig. 1:**
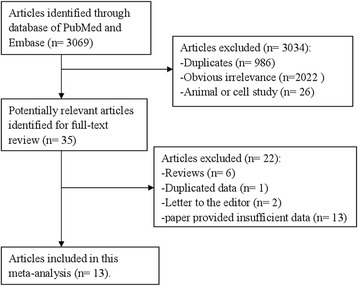
Flow diagram of the literature search and trials selection process

We have searched databases of PubMed and Embase and finally got 3069 articles. We then reviewed the titles and abstracts to exclude 3034 articles because of duplicates (*n* = 986), obvious irrelevance (*n* = 2022), and animal or cell studies (*n* = 26). Twenty-two of the remaining 35 articles are excluded due to the following reasons: review articles (*n* = 6), duplicate publications (*n* = 1), letter to the editors (*n* = 2), and paper provided insufficient data (*n* = 13). Finally, 13 articles [7–9, 17–26] were included in this study. Two articles [22, 25] reported colon cancer and rectum cancer, two articles [23, 24] reported male populations and female populations, one article [26] reported Whites and African Americans, and one article [9] reported animal protein and vegetable protein, respectively. Therefore, 21 studies involving 8187 cases were used for the analysis. And the screening process was showed by Fig. 1. The average NOS score was 7.23, and all included studies were with high quality (over six stars). The details of the quality score for every study are shown in Additional file 1: Table S1. The characteristics of included studies are listed in Table 1.

**Table 1:** Characteristics of the included studies

Study, year	Country	Study design	Participants (cases)	Age (years)	Score quality	Protein type	Dietary assessment	Protein intake amounts (g)	RR (95% CI)	Adjustment for covariates
Ghadirian P, 1997	Canada	Case-control	1070 (402)	35–79	6	Total animal vegetable	Quantitative food frequency questionnaire	Colon Quartile 1 Quartile 2 Quartile 3 Quartile 4	Colon 1 0.81(0.55–1.19) 0.91(0.62–1.32) 1.11(0.77–1.61)	Adjusted for gender, age, marital status, history of colon carcinoma in first-degree relatives, and total energy intake
Goldbohm RA, 1994	Netherlands	Cohort	120,852 (215)	55–69	8	Animal	Semiquantitative food frequency questionnaire	Colon Quintile 1 Quintile 2 Quintile 3 Quintile 4 Quintile 5	Colon 1 1.10(0.70–1.71) 1.05(0.67–1.65) 1.28(0.82–2.00) 0.90(0.57–1.42)	Adjusted for quintiles of energy and energy-adjusted nutrient intakes, sex, and dietary fiber intake
Iscovich JM, 1992	Argentina	Case-control	330 (110)	NA	7	Total	Food-frequency questionnaire	Colon Quartile 1 Quartile 2 Quartile 3 Quartile 4	Colon 1 0.94(0.46–1.90) 0.63(0.26–1.49) 1.03(0.33–3.21)	Adjusted for fiber at 19.02 g per day, other sources of energy intake
Levi F, 2002	Switzerland	Case-control	836 (286)	≤ 74	7	Total	Food-frequency questionnaire	Colorectal Lowest Middle Highest	Colorectal 1 0.80(0.50–1.30) 1.10(0.70–1.70)	Adjusted for age, sex, education, physical activity, and residual energy
Pietinen P, 1999	Finland	Cohort	27,111 (185)	50–69	8	Total Animal Vegetable	Food-frequency questionnaire	Colorectal 83.8 96.9 105.1 115.9	Colorectal 1 1.20(0.80–1.70) 0.90(0.60–1.40) 0.90(0.50–1.50)	Adjusted for age, supplement group, smoking years, body mass index, alcohol, education, and physical activity at work, and calcium intake (except for milk protein and milk products)
Prentice RL, 2009	American	Cohort	59,105 (280)	50–89	8	Total	Food-frequency questionnaire	Colon Quartile 1 Quartile 2 Quartile 3 Quartile 4	Colon 1 0.97(0.70–1.34) 1.11(0.76–1.61) 0.96(0.64–1.44)	NA
Prentice RL, 2009	American	Cohort	59,105 (280)	50–89	8	Total	Food-frequency questionnaire	Rectum Quartile 1 Quartile 2 Quartile 3 Quartile 4	Rectum 1 1.22(0.56–2.65) 1.57(0.72–3.41) 1.08 (0.48–2.41)	NA
Slattery ML, 1994	American	Case-control	623 (231)	40–79	7	Total	Food-frequency questionnaire	Colon ≤ 58.7 58.8–81.4 81.5–99.9 > 99.9	Colon (men) 1 2.30(1.10–4.70) 2.40(1.00–5.70) 3.70(1.30–10.50)	Adjusted for age, religion, body mass index, crude fiber, and separate logistic models were used for each variable. Calcium as continuous variables in multiple logistic regression models.
Slattery ML, 1994	American	Case-control	623 (231)	40–79	7	Total	Food-frequency questionnaire	Colon ≤ 49.7 49.8–63.5 63.6–79.1 > 79.1	Colon (women) 1 0.90(0.40–1.90) 1.90(0.90–4.20) 2.70(0.90–7.70)	Adjusted for age, religion, body mass index, crude fiber, and separate logistic models were used for each variable. Calcium as continuous variables in multiple logistic regression models
Slattery ML, 1997	American	Case-control	2389 (1099)	30–79	7	Total	Food-frequency questionnaire	Colon (kcal) ≤ 263 264–331 332–407 408–512 > 512	Colon (men) 1 1.25(0.95–1.65) 1.35(1.01–1.81) 1.33(0.95–1.84) 1.73(1.11–2.68)	Adjusted for age, body mass index, and family history of first-degree relative with colorectal cancer, use of aspirin and/or nonsteroidal anti-inflammatory drugs, physical activity, and dietary intake of fiber, cholesterol, and calcium
Slattery ML, 1997	American	Case-control	2014 (894)	30–79	7	Total	Food-frequency questionnaire	Colon (kcal) ≤ 209 210–259 260–316 317–399 > 399	Colon (women) 1 1.00(0.74–1.35) 1.02(0.74–1.40) 1.00(0.70–1.43) 0.91(0.56–1.48)	Adjusted for age, body mass index, and family history of first-degree relative with colorectal cancer, use of aspirin, and/or nonsteroidal anti-inflammatory drugs, physical activity, and dietary intake of fiber, cholesterol, and calcium
Sun Z, 2012	Canada	Case-control	4241 (1760)	20–74	7	Total	Food-frequency questionnaire	Colorectal Quintile 1 Quintile 3 Quintile 5	Colorectal 1 0.88(0.70–1.11) 0.85(0.69–1.00)	Adjusted for total energy intake. Other potential confounders included age, sex, BMI, physical activity, family history of CRC, polyps, diabetes, reported colon screening procedure, cigarette smoking, alcohol drinking, education attainment, household income, marital status, regular use of NSAID, regular use of multivitamin supplements, regular use of folate supplement, regular use of calcium supplement, reported HRT (females only), province of residence, and intakes of fruits, vegetables, and red meat. Variables were included in the final model based on *a* ≥ 10% alternation in the parameter coefficient of interest
Tayyem RF, 2015	Jordan	Case-control	417 (169)	53.8 ± 12.2	6	Total	Quantitative food frequency questionnaire	Colorectal Quartile 1 Quartile 2 Quartile 3 Quartile 4	Colorectal 1 1.66(0.74–3.69) 1.74(0.78–3.90) 3.62(1.63–8.04)	Adjusted for total energy intake normality of the distributions of dietary intake variables was assessed by the Shapiro-Wilk test. Non-normally distributed variables were log transformed. Other potential confounders included age, gender, BMI, physical activity (METs/week), family history (beyond the second degree) of CRC, education attainment, household income, marital status, and tobacco use
Wakai K, 2006	Japan	Case-control	3042 (507)	20–79	7	Total	Food-frequency questionnaire	Colon Quartile 1 Quartile 2 Quartile 3 Quartile 4	Colon 1 0.74(0.52–1.07) 0.65(0.45–0.95) 0.74(0.51–1.07)	Adjusted for age, sex, year of first visit, season of first visit to the hospital, reason for the visit, family history of colorectal cancer, body mass index, exercise, alcohol drinking, smoking, multivitamin use, and energy intake
Wakai K, 2006	Japan	Case-control	3042 (507)	20–79	7	Total	Food-frequency questionnaire	Rectum Quartile 1 Quartile 2 Quartile 3 Quartile 4	Rectum 1 0.83(0.57–1.22) 0.91(0.62–1.32) 0.71(0.47–1.06)	Adjusted for age, sex, year of first visit, season of first visit to the hospital, reason for the visit, family history of colorectal cancer, body mass index, exercise, alcohol drinking, smoking, multivitamin use, and energy intake
Williams CD, 2010	American	Case-control	1520 (720)	59.6 ± 10.3	8	Total	Food-frequency questionnaire	Colorectal Quartile 1 Quartile 2 Quartile 3 Quartile 4	Colorectal (whites) 1 0.90(0.64–1.27) 0.86(0.57–1.30) 0.57(0.32–1.01)	Adjusted for age, sex, education, body mass index, family history, nonsteroidal anti-inflammatory drug use, physical activity, calcium, fiber, and total energy
Williams CD, 2010	American	Case-control	384 (225)	58.0 ± 10.0	8	Total	Food-frequency questionnaire	Colorectal Quartile 1 Quartile 2 Quartile 3 Quartile 4	Colorectal (African Americans) 1 1.05(0.51–2.16) 0.81(0.32–2.05) 0.58(0.16–2.10)	Adjusted for age, sex, education, body mass index, family history, nonsteroidal anti-inflammatory drug use, physical activity, calcium, fiber, and total energy
Yang SY, 2016	Korea	Case-control	1056 (406)	30–70	8	Animal	Food-frequency questionnaire	Colorectal Tertile 1 Tertile 2 Tertile 3	Colorectal (men) 1 0.71(0.47–1.07) 1.25(0.88–1.78)	Adjusted for total energy intake, waist circumference, BMI, HDL cholesterol, fasting glucose, alcohol intake, smoking status, and family history of colorectal adenoma
Yang SY, 2016	Korea	Case-control	658 (151)	30–70	8	Animal	Food-frequency questionnaire	Colorectal Tertile 1 Tertile 2 Tertile 3	Colorectal (women) 1 0.71(0.30–1.68) 0.87(0.52–1.46)	Adjusted for total energy intake, waist circumference, BMI, HDL cholesterol, fasting glucose, alcohol intake, smoking status, and family history of colorectal adenoma
Yang SY, 2016	Korea	Case-control	1056 (406)	30–70	8	Vegetable	Food-frequency questionnaire	Colorectal Tertile 1 Tertile 2 Tertile 3	Colorectal (men) 1 0.93(0.63–1.37) 0.96(0.60–1.53)	Adjusted for total energy intake, waist circumference, BMI, HDL cholesterol, fasting glucose, alcohol intake, smoking status, and family history of colorectal adenoma
Yang SY, 2016	Korea	Case-control	658 (151)	30–70	8	Vegetable	Food-frequency questionnaire	Colorectal Tertile 1 Tertile 2 Tertile 3	Colorectal (women) 1 0.78(0.39–1.56) 0.54(0.27–1.11)	Adjusted for total energy intake, waist circumference, BMI, HDL cholesterol, fasting glucose, alcohol intake, smoking status, and family history of colorectal adenoma

Abbreviations: *NA* not available, *BMI* body mass index, *CRC* colorectal cancer, *CI* confidence interval, *RR* relative risk

### Dietary protein intake and risk of colorectal cancer

**Fig. 2:**
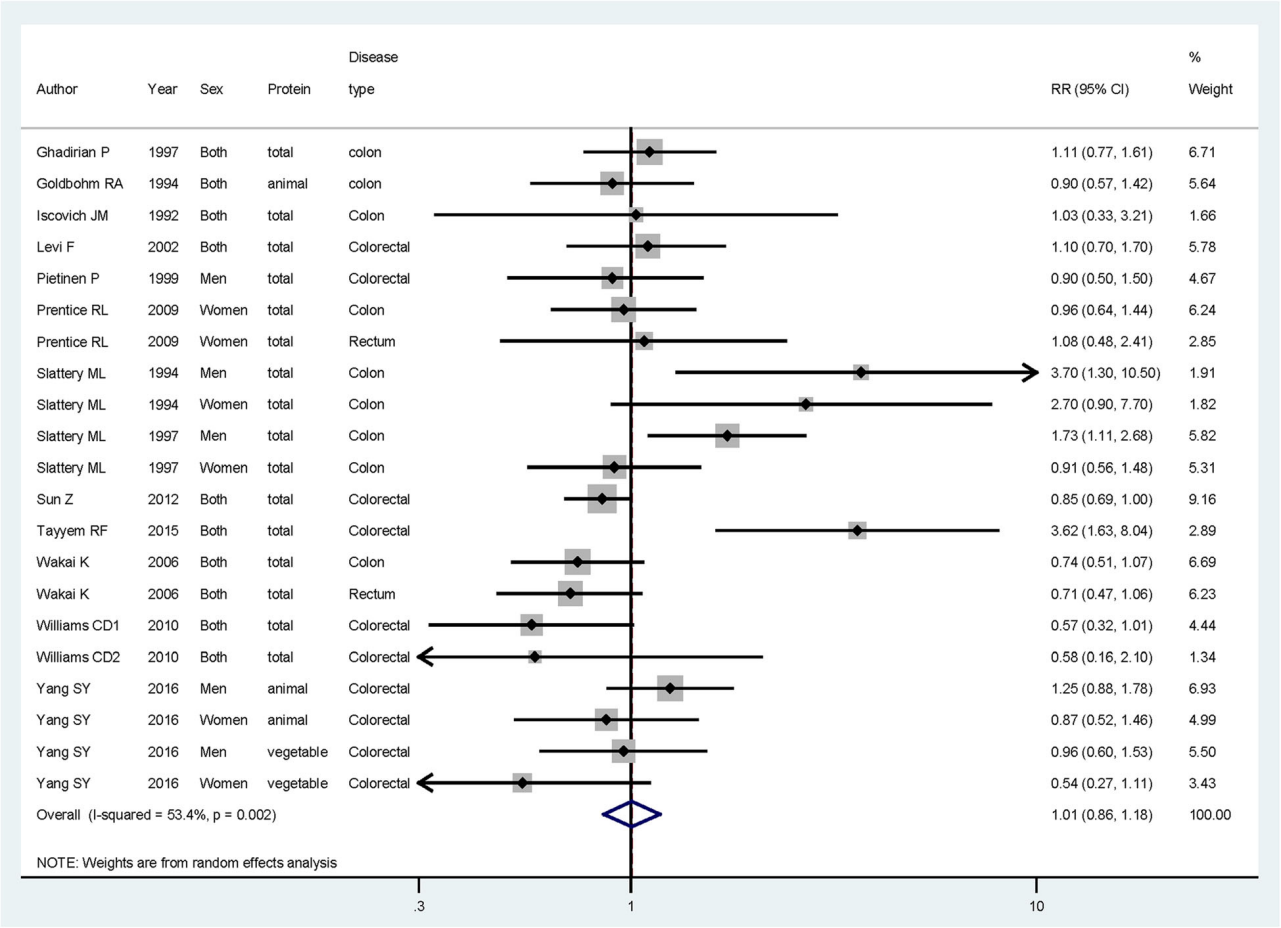
The forest plot between dietary protein intake and colorectal cancer risk

To assess if the intake of protein could influence the colorectal cancer risk, we used random-effect model to pool the study-specific RR. Compared with the lowest category of protein intake, the pooled RR of colorectal cancer for the highest category was 1.006 (95% CI = 0.857–1.179) indicating that highest category of dietary protein intake had no significant association on colorectal cancer risk (Fig. 2). Heterogeneity was found to be significant (*I* ^*2*^ = 53.4%, *p* = 0.002).

### Sources of heterogeneity and subgroup analysis

In the pooled results of the overall studies, significant heterogeneity was demonstrated for the associations between dietary protein intake and colorectal cancer risk. Thus, we used univariate meta-regression to explore the reason of causing with the covariates of publication year, disease type, study design, protein type (animal protein or vegetable protein), sex, and geographic locations. No significant findings were found contributing significantly to the between-study heterogeneity.

**Fig. 3:**
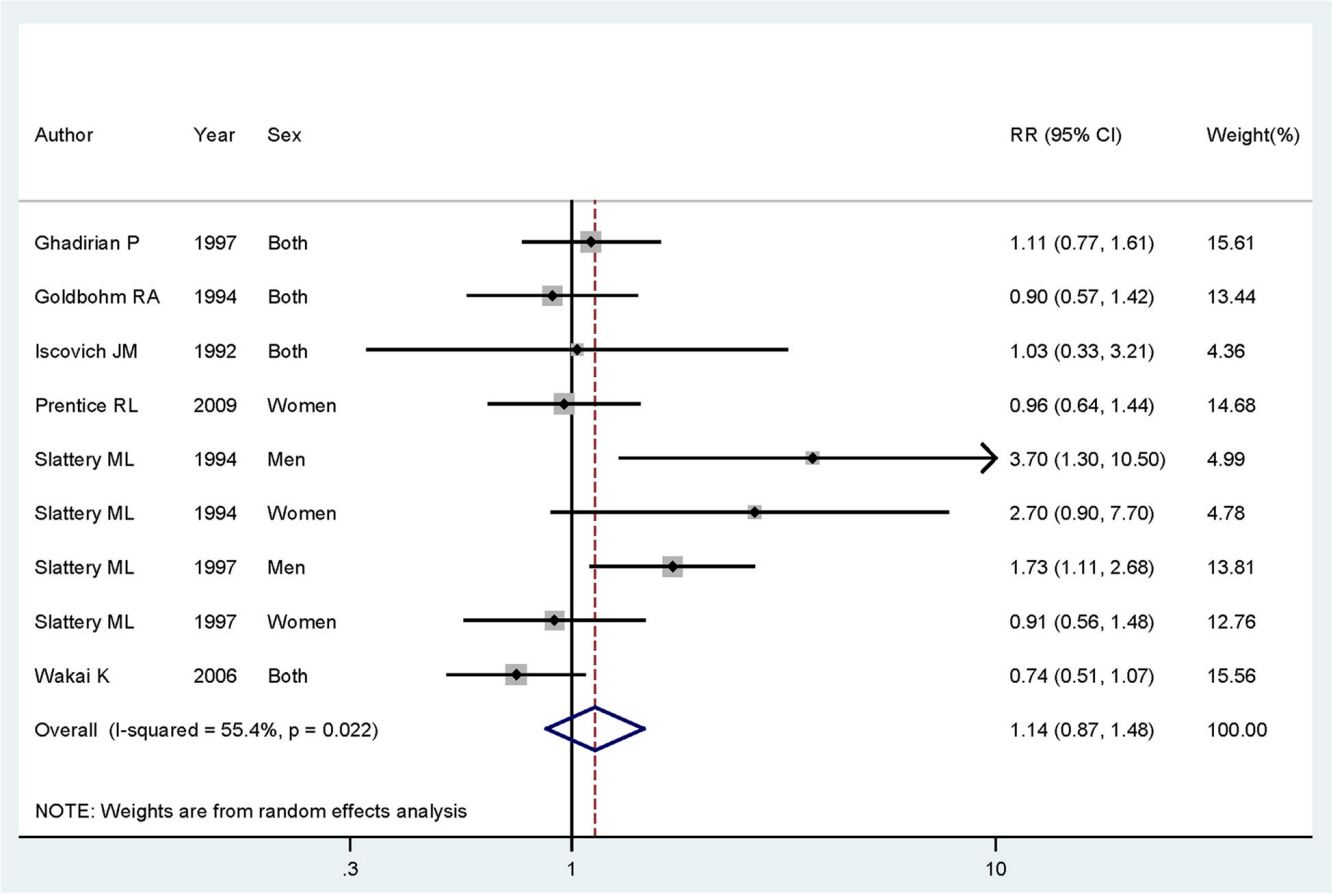
The forest plot between the highest versus the lowest categories of dietary protein intake and colon cancer risk

Subgroup analyses were also conducted to explore the potential relationship and heterogeneity. For the subgroup of disease type, the pooled RRs for colon cancer and rectum cancer were 1.135 (95% CI = 0.871–1.480) (Fig. 3) and 0.773 (95% CI = 0.538–1.111) with the highest category of dietary protein intake, respectively. As different types of proteins could influence the risk of colorectal cancer, a subgroup analysis has been conducted. The results showed that no evidence of significance was found between dietary animal protein or vegetable protein intake and colorectal cancer risk. When we conducted the subgroup analysis by study design, the highest dietary protein intake levels had nonsignificant association for colorectal cancer risk either in cohort studies or in case-control studies. In a stratified analysis by sex and geographic locations, the association was consistent with the overall result. The detailed results are shown in Table 2.

**Table 2:** Combined overall and subgroup results

Subgroups	No. cases	No. studies	RR (95% CI)	*I* ^2^ (%)	*P*_heterogeneity_
All studies	8187	21	1.006(0.857–1.179)	53.4	0.002
Disease type					
Colon	3446	9	1.135(0.871–1.480)	55.4	0.022
Rectum	282	2	0.773(0.538–1.111)	0.0	0.363
Study design					
Cohort	680	4	0.939(0.730–1.209)	0.0	0.980
Case-control	7507	17	1.030(0.846–1.254)	62.6	0.000
Sex					
Both	4394	10	0.913(0.737–1.132)	53.3	0.023
Men	2204	5	1.306(0.932–1.829)	54.6	0.066
Women	1589	6	0.931(0.710–1.220)	20.5	0.279
Protein					
Total	6858	16	1.049(0.856–1.285)	60.6	0.001
Animal	1359	5	1.041(0.866–1.252)	0.0	0.624
Vegetable	1144	4	0.851(0.602–1.203)	49.9	0.112
Geographic locations					
America	5711	11	1.072(0.838–1.372)	56.4	0.011
Asia	1790	7	0.962(0.694–1.333)	69.0	0.004
Europe	686	3	0.972(0.738–1.280)	0.0	0.785

### Sensitive analysis

In a sensitivity analysis excluding a single study at a time, no individual study would affect the whole result.

### Publication bias

**Fig. 4:**
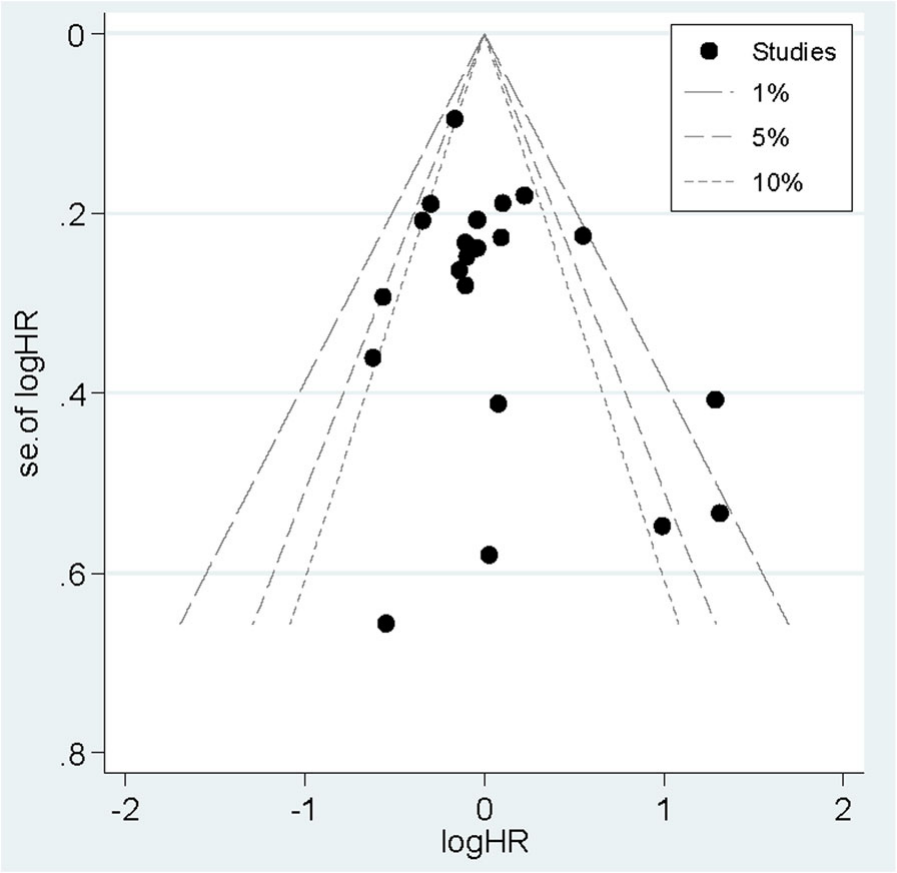
The funnel plot of the association between dietary protein intake and colorectal cancer risk

The Egger’s test (*P* = 0.201) and Begg’s funnel plot (Fig. 4) detected no obvious publication bias.

## Discussion

Findings from this report of observational studies indicated that no evidence of significant association was found on colorectal cancer risk with the highest category of dietary protein intake. The association was not significant either in the colon cancer risk or in the rectum cancer risk with the highest category of dietary protein intake. The results of subgroup analyses by study design, protein type, sex, and geographic locations were consistent with the overall pooled result. To our knowledge, this is the first comprehensive report conducted to assess the relation between dietary protein intake and colorectal cancer risk. Although we did not find any positive result between them, a large number of colorectal cancer cases could allow a much greater possibility of reaching reasonable conclusions.

Significant between-study heterogeneity was found in the process of merging results (*I* ^2^ = 53.4%, *P*_heterogeneity_ = 0.002). However, heterogeneity is common in the meta-analysis [27]. Meta-regression was used to explore the potential covariates that cause between-study heterogeneity. All the covariates of publication year, disease type, study design, protein type, sex, and geographic locations were not found any significant contributed to the heterogeneity. Furthermore, subgroup analyses by disease type, study design, protein type, sex, and geographic locations were performed to explore the high heterogeneity. To our attention, the heterogeneity was not significant either in the animal protein studies (*I* ^2^ = 0.0%, *P*_heterogeneity_ = 0.624) or vegetable protein studies (*I* ^2^ = 49.9%, *P*_heterogeneity_ = 0.112). Therefore, the protein type may increase the heterogeneity to the whole results.

To our attention, various studies showed an opposite correlation (some positive and others inverse) between dietary intake of protein and colorectal cancer. The methods of dietary assessment in all the included studies are food frequency questionnaire (FFQ). Subgroup analysis by geographic locations was conducted to find the opposite correlation between dietary intake of protein and colorectal cancer, but there is no significant association among American populations, Asian populations, or European populations. The detailed amounts of dietary protein intake are not provided in most studies. Then, we did not consider the influences of dietary intake considerations to the colorectal cancer risk. Furthermore, the results are unstable due to the small sample size in each independent study. Therefore, this meta-analysis was performed to obtain a comprehensive result of this issue.

Several restrictions in this report should be attended. Firstly, our study included 21 individual studies; therefore, many diversification such as adjustments and quality that may influence the whole result. Secondly, although most of the included studies have adjusted for potential confounding factors, such as age, sex, and BMI, we cannot rule out the possibility that some other un-measured factors, such as intake of other energy, might have been partly responsible for the observed association. Thirdly, due to the limited information in the reported studies, dose-response analysis was not performed for dietary protein intake and colorectal cancer risk. Therefore, detailed information for each category of protein intake is wanted to assess the relationship between protein intake and colorectal cancer. Fourthly, dietary protein intake was collected via food-frequency questionnaires (FFQs), recall bias, and measurement bias could not be eliminated. Finally, significant heterogeneity was observed across studies included in the present meta-analysis. And the between-study heterogeneity was not resolved by meta-regression, the results of subgroup analyses were also with high heterogeneity in some groups except in the subgroup of protein type. Therefore, dietary protein type (animal protein or vegetable protein) may be the potential contributor to the between-study heterogeneity, and it would be further affecting the heterogeneity.

## Conclusions

In summary, this study suggested that the highest category of dietary protein intake had no significant association for the risk of colorectal cancer. Therefore, further studies with large cases and participants as well as detailed amounts of dietary protein intake are wanted to confirm this result, during some limitation that existed in our study.

## Additional file

Additional file 1: Table S1. The details of quality score for the included study. (DOCX 12 kb)

## Abbreviations

CI: Confidence intervals
HR: Hazard ratio
NOS: Newcastle-Ottawa Scale
OR: Odds ratio
PRISMA: Preferred Reporting Items for Systematic Reviews and Meta-Analyses
RR: Risk ratio
