# Supplementary material for: The association between dietary protein intake and colorectal cancer risk: a meta-analysis
Source: World J Surg Oncol. 2017 Sep 8;15:169. doi: 10.1186/s12957-017-1241-1 (PMC5591555; doi:10.1186/s12957-017-1241-1)
Supplement: Additional file 1: Table S1. — The details of quality score for the included study. (DOCX 12 kb) [file 12957_2017_1241_MOESM1_ESM.docx]

Table S1 The details of quality score for the included study.

| Study | Selection | Comparability | Exposure | Score quality |
| --- | --- | --- | --- | --- |
| Ghadirian P, 1997 | ☆☆☆ | ☆ | ☆☆ | 6 |
| Goldbohm RA, 1994 | ☆☆☆☆ | ☆☆ | ☆☆ | 8 |
| Iscovich JM, 1992 | ☆☆☆☆ | ☆ | ☆☆ | 7 |
| Levi F, 2002 | ☆☆☆☆ | ☆ | ☆☆ | 7 |
| Pietinen P, 1999 | ☆☆☆☆ | ☆☆ | ☆☆ | 8 |
| Prentice RL, 2009 | ☆☆☆☆ | ☆☆ | ☆☆ | 8 |
| Slattery ML, 1994 | ☆☆☆☆ | ☆ | ☆☆ | 7 |
| Slattery ML, 1997 | ☆☆☆☆ | ☆ | ☆☆ | 7 |
| Sun Z, 2012 | ☆☆☆☆ | ☆ | ☆☆ | 7 |
| Tayyem RF, 2015 | ☆☆☆ | ☆ | ☆☆ | 6 |
| Wakai K, 2006 | ☆☆☆☆ | ☆ | ☆☆ | 7 |
| Williams CD, 2010 | ☆☆☆☆ | ☆☆ | ☆☆ | 8 |
| Yang SY, 2016 | ☆☆☆☆ | ☆☆ | ☆☆ | 8 |
